# Impact of Climate Change on Reproductive Health and Pregnancy Outcomes: A Systematic Review

**DOI:** 10.7759/cureus.68221

**Published:** 2024-08-30

**Authors:** Aggeliki Papadiochou, Athina Diamanti, Dimitra Metallinou, Vasiliki E Georgakopoulou, Chrysoula Taskou, Iraklis Kagkouras, Antigoni Sarantaki

**Affiliations:** 1 Department of Midwifery, University of West Attica, Athens, GRC; 2 Department of Pathophysiology/Pulmonology, Laiko General Hospital, Athens, GRC; 3 Department of Surgery, London Hospital, London, GBR

**Keywords:** environmental exposure, air pollution, pregnancy outcomes, reproductive health, climate change

## Abstract

Climate change has emerged as a significant global health challenge, with growing evidence linking environmental factors to adverse reproductive health outcomes. The primary objective of this review is to assess the effects of climate change-driven environmental factors, such as air pollution and temperature extremes, on reproductive health outcomes, including fertility rates, miscarriage, preterm birth, and congenital anomalies. A comprehensive search of PubMed, Google Scholar, and Web of Science was conducted until July 2024. Studies included in the review were observational, experimental, and randomized controlled trials that reported quantitative data on reproductive outcomes in relation to climate-related environmental exposures. A total of 49 studies were selected for qualitative synthesis. The review found that increased exposure to particulate matter (PM2.5), extreme temperatures, and proximity to traffic were consistently associated with reduced fertility, increased risks of miscarriage, preterm birth, and low birth weight. Adverse effects were particularly pronounced among vulnerable populations, such as pregnant women of lower socioeconomic status and those living in disaster-prone areas. The studies also highlighted potential transgenerational effects, with prenatal exposure to environmental stressors influencing the long-term health of offspring. The findings underscore the urgent need for public health interventions and policies to mitigate environmental exposures that negatively impact reproductive health. Future research should focus on longitudinal and interventional studies to establish causal relationships and inform effective public health strategies.

## Introduction and background

Increasing global temperatures due to climate change are leading to various environmental issues such as droughts, wildfires, worsening air quality, and unpredictable weather patterns. These factors collectively contribute to a global health crisis [1]. The rise in industrial activities worldwide has amplified the burning of fossil fuels, resulting in elevated levels of greenhouse gases, which are major contributors to the climate crisis [2]. According to projections by the World Health Organization, an estimated 250,000 additional deaths may occur due to climate-sensitive diseases between 2030 and 2050 [3]. Recent years have witnessed numerous severe climate and environmental disasters. In 2020 alone, 132 natural disasters, including floods, storms, and droughts, affected 51.6 million people and caused 3,000 deaths [4]. The wildfires in the United States during 2020 impacted 2.3 million individuals [4].

Global warming and frequent flooding events have also been linked to an increase in the transmission of vector-borne diseases, such as Zika virus (ZIKV) and malaria [5]. These climate-related health impacts disproportionately affect vulnerable populations, including pregnant individuals and developing fetuses, who often have limited access to healthcare and resources. Such populations face challenges like displacement from their homes, food scarcity, and exposure to poor air quality. A notable example is Hurricane Sandy, which struck New York in October 2012. It caused one of the largest power outages in U.S. history and resulted in significant increases in pregnancy complications among Black and Hispanic individuals (20.9% and 25.9%, respectively) compared to a 5.6% increase among White individuals [6]. The stress from this event was found to have transgenerational effects, with higher prenatal maternal stress correlating with altered infant temperament at six months of age [7].

Additionally, global trends over the past 50 years indicate a decline in fecundity, characterized by decreasing sperm counts, and increasing rates of miscarriage and preterm births [8-11]. Even after accounting for factors such as age and socioeconomic status, these trends remain significant and are observed globally. Experts generally agree that environmental factors, including climate change, play a role in these reproductive health issues. This review explores the impacts of climate change on reproductive health and pregnancy outcomes.

## Review

Search strategy and selection criteria

This systematic review was conducted to assess the impact of environmental factors, including climate change, on reproductive health and pregnancy outcomes. A comprehensive search strategy was employed using the databases PubMed, Google Scholar, and Web of Science till July 2024. The search terms included combinations of "climate change," "global warming," "fertility," "reproductive health," "pregnancy outcomes," "air pollution," "particulate matter," "temperature extremes," "heat stress," "miscarriage," "pregnancy loss," "preterm birth," "low birth weight," "infertility," "IVF outcomes," "maternal exposure," "environmental toxins," "wildfire smoke," "ozone," "PM2.5," "PM10," "NO2," "endocrine disruption," "placental function," "birth defects," and "congenital anomalies." The inclusion criteria were limited to studies published in English, involving mammalian species, and available as full-text articles.

Inclusion criteria

(a) Observational studies, randomized controlled trials (RCTs), and experimental animal studies evaluating the impact of environmental exposures on reproductive outcomes; (b) studies providing quantitative data on reproductive outcomes such as fertility rates, pregnancy rates, miscarriage, preterm birth, and low birth weight; and (c) peer-reviewed articles with clear definitions of environmental exposures and reproductive health outcomes were included in this study.

Exclusion criteria

(a) Studies lacking specific details on environmental exposures or reproductive health outcomes; (b) non-peer-reviewed articles, reviews, editorials, case reports, and letters; and (c) studies focusing solely on occupational exposures without broader environmental relevance were excluded from this study.

PRISMA process

**Figure 1:**
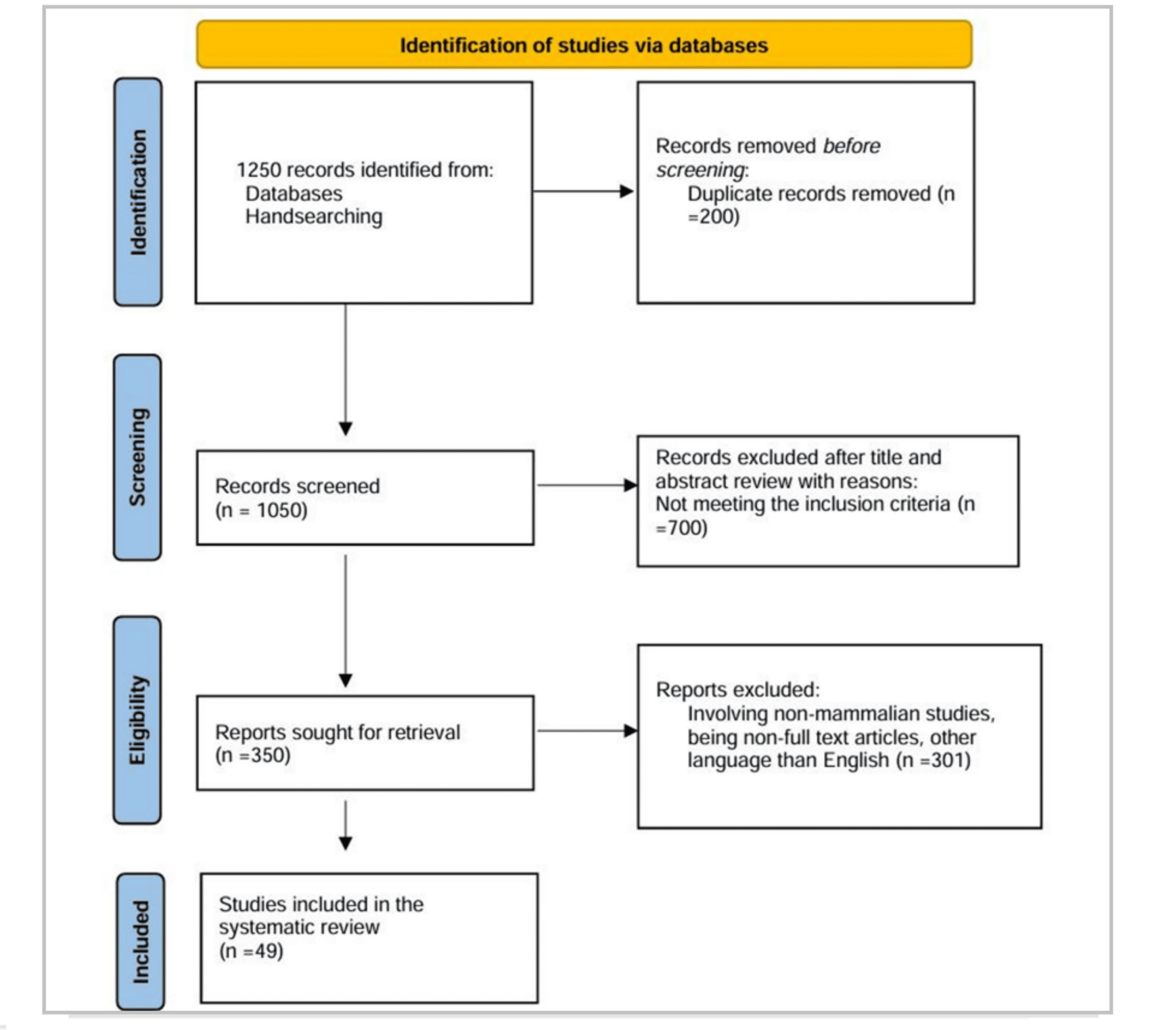
PRISMA flowchart for the selection of studies

The systematic review adhered to the PRISMA (Preferred Reporting Items for Systematic Reviews and Meta-Analyses) guidelines. The PRISMA flow diagram (Figure 1) documented the process of study selection, beginning with the initial identification of studies through database searches and ending with the inclusion of studies in the qualitative synthesis.

Identification

A total of 1200 records were identified through database searching. In addition, 50 more records were identified through manual hand-searching.

Screening

After removing duplicates, 1050 records were screened based on titles and abstracts, and 700 records were excluded as they did not meet the inclusion criteria.

Eligibility

The remaining 350 full-text articles were assessed for eligibility, with 301 articles excluded due to several reasons such as not being in English, involving non-mammalian studies, or being non-full-text articles.

Included studies

Ultimately, 49 studies met all the criteria and were included in the qualitative synthesis.

Data extraction and management

Data extraction was performed independently by two reviewers using a standardized data extraction form. The extracted data included study characteristics (authors, year, location, and study design), exposure details (type, duration, and measurement methods), outcome measures (reproductive outcomes, definitions, and assessment tools), and key findings (results, statistical significance, effect sizes). Discrepancies between reviewers were resolved through discussion, with a third reviewer consulted as necessary.

Quality assessment

The quality of included studies was assessed using different standardized tools based on study design. The Newcastle-Ottawa Scale (NOS) was used for observational studies, focusing on selection, comparability, and outcome ascertainment. Studies were rated on a scale, with higher scores indicating better quality. The Cochrane Risk of Bias Tool for RCTs was employed to evaluate domains such as random sequence generation, allocation concealment, blinding, incomplete outcome data, and selective reporting. The SYRCLE's (Systematic Review Centre for Laboratory Animal Experimentation) Risk of Bias tool was used to assess bias in animal studies, covering aspects like sequence generation, baseline characteristics, allocation concealment, blinding, random housing, and outcome assessment.

Studies were categorized as having a low, moderate, or high risk of bias. Sensitivity analyses were performed to assess the impact of excluding studies with a high risk of bias on the overall conclusions of the review.

Results

The systematic review included a total of 49 studies, encompassing various research designs such as prospective cohort studies, RCTs, case-control studies, cross-sectional studies, and experimental animal studies [12-60]. The included studies were conducted across different geographic regions and investigated a wide range of environmental exposures and reproductive health outcomes.

Impact of air pollution

Proximity to Major Roadways and IVF Success

Gaskins et al. [12] found that women living closer to major roadways had a lower probability of successful implantation and live birth during in vitro fertilization (IVF). Mendola et al. [17] observed longer times to pregnancy and higher infertility risks among couples living closer to major roadways, although these associations were not statistically significant. Quraishi et al. [23] reported a significant decrease in live birth rates among women residing near major roadways, particularly those with specific infertility diagnoses like diminished ovarian reserve.

Particulate Matter and Ovarian Function

Gaskins et al. [13] demonstrated that higher exposure to particulate matter 2.5 micrometers or less in diameter (PM2.5) was associated with reduced ovarian reserve, as indicated by lower antral follicle counts (AFC). Ogliari et al. [21] showed that intrauterine exposure to diesel exhaust PM2.5 reduced ovarian reserve in mice, suggesting potential long-term impacts on reproductive lifespan.

Air Pollution and IVF Success

Legro et al. [15] found that nitrogen dioxide (NO_2_) and PM2.5 exposure negatively affected IVF outcomes, particularly in reducing live birth rates. PM2.5 exposure during embryo culture was linked to lower conception rates. Perin et al. [22] noted that while particulate matter 10 micrometers or less in diameter (PM10) exposure during the follicular phase did not significantly impact IVF clinical outcomes, it was associated with an increased risk of miscarriage.

General Fertility and Fecundability

Slama et al. [24] and Wesselink et al. [26] found that higher exposure to PM2.5 and NO_2_ was associated with reduced fecundability, lowering the likelihood of conception within a specific timeframe.

Impact of Air Pollution on Sperm Quality and Male Fertility

Hammoud et al. [14] reported that higher PM2.5 levels were associated with decreased sperm motility in men, which could negatively impact male fertility. Yang et al. [27] found that chronic exposure to diesel exhaust PM2.5 impaired spermatogenesis in mice, leading to reduced sperm count and motility. Chen et al. [33] observed that higher exposure to sulfur dioxide (SO_2_) and nitrogen oxides (NOx) was linked to decreased testicular volume and impaired sperm quality in infertile men.

Impact of Air Pollution on Preterm Birth and Birth Defects

Zhu et al. [28] found that higher exposure to PM2.5 during pregnancy was associated with an increased risk of preterm birth. Kwag et al. [51] showed that heat waves combined with high PM2.5 levels significantly increased the risk of preterm birth in Korea. Cheng et al. [32] linked higher concentrations of PM10, PM2.5, NO_2_, and SO_2_ during the first trimester to an increased risk of birth defects, particularly congenital heart defects. Padula et al. [30] found that exposure to airborne polycyclic aromatic hydrocarbons (PAHs) during the last six weeks of pregnancy significantly increased the risk of early preterm birth, while a complex relationship was observed between PAH exposure and preterm birth risk during earlier pregnancy stages.

Impact of Air Pollution on Low Birth Weight and Placental Function

Veras et al. [25] demonstrated that exposure to urban air pollution in mice led to smaller fetal weights and adverse changes in placental morphology, potentially contributing to intrauterine growth restriction (IUGR). Díaz et al. [31] found that PM2.5 exposure during the third month of pregnancy was associated with an increased risk of low birth weight (LBW) among non-premature infants. Padula et al. [29] reported that higher traffic density near maternal residences during pregnancy was associated with a higher probability of term LBW, with the strongest associations observed near high-volume freeways.

Impact of Air Pollution on Missed Abortion in the First Trimester (MAFT)

Zhang et al. [40] found a significant association between higher levels of air pollution and an increased risk of MAFT in Beijing, China. Higher concentrations of PM2.5, SO_2_, O_3_ (ozone), and carbon monoxide (CO) were linked to a higher risk of MAFT, with more severe risks observed as pollutant concentrations increased.

Traffic-Related Pollution and Urban Fertility

Nieuwenhuijsen et al. [20] found that higher levels of coarse particulate matter (PMcoarse), a component of traffic-related air pollution, were associated with lower fertility rates in urban areas of Barcelona, Spain.

Diesel Exhaust and Embryo Development

Januário et al. [19] showed that diesel exhaust particles impaired early embryo development in mice, reducing blastocyst formation and increasing apoptosis, suggesting risks to reproductive health from diesel exhaust exposure. Table 1 summarizes the studies regarding the impact of air pollution.

**Table 1:** Summary of studies included in the systematic review regarding the impact of air pollution AFC: Antral follicle count; AQI: Air quality index; CHDs: Congenital heart defects; CO: Carbon monoxide; Cdx-2: Caudal type homeobox 2; DEP: Diesel exhaust particles; DSBs: Double-strand breaks; ET: Embryo transfer; IVF: In vitro fertilization; LBW: Low birth weight; Leqd: Equivalent continuous noise level (day); Leqn: Equivalent continuous noise level (night); NO_2_: Nitrogen dioxide; NOx: Nitrogen oxides; Oct-4: Octamer-binding transcription factor 4; O_3_: Ozone; PAHs: Polycyclic aromatic hydrocarbons; PM: Particulate matter; PM2.5: Particulate matter with a diameter of 2.5 micrometers or less; PM10: Particulate matter with a diameter of 10 micrometers or less; PMcoarse: Coarse particulate matter; SO_2_: Sulfur dioxide.

Authors	Year	Type of study	Sample	Measurements	Outcomes	Key findings
Gaskins et al. [12]	2018	Prospective cohort	423 women, 726 IVF cycles	Residential proximity to roadways, traffic density, IVF outcomes (implantation, live birth), estradiol levels, and endometrial thickness	Implantation, clinical pregnancy, live birth, estradiol levels, and endometrial thickness	Closer residential proximity to major roadways was significantly associated with a lower probability of implantation and live birth following IVF. Traffic density was not associated with IVF outcomes.
Gaskins et al. [13]	2019	Prospective cohort	632 women from a fertility clinic	Residential PM2.5 exposure (estimated for 3 months before antral follicle count) and antral follicle count (AFC)	Antral follicle count, a marker of ovarian reserve	Higher exposure to PM2.5 was associated with a 7.2% lower AFC, suggesting that air pollution may accelerate reproductive aging, particularly among women with female factor infertility.
Hammoud et al. [14]	2010	Ecological study	1,699 semen analyses and 877 inseminations	PM2.5 levels, sperm concentration, motility, and morphology	Sperm motility and morphology	Higher PM2.5 levels correlated with reduced sperm motility, particularly two to three months after exposure. No significant correlation was found with sperm concentration.
Legro et al. [15]	2010	Observational study	7,403 females undergoing IVF	Air quality data (NO_2_, O_3_, PM2.5, PM10, and SO_2_) and IVF outcomes (pregnancy and live birth rates)	Pregnancy and live birth rates	Higher NO_2_ levels were consistently associated with lower live birth rates. Ozone showed mixed effects, with increased live birth rates during ovulation induction and decreased rates post-embryo transfer. PM2.5 at IVF labs was linked to reduced conception rates.
Mahalingaiah et al. [16]	2016	Prospective cohort	36,294 women from the Nurses' Health Study II	Residential proximity to major roadways, exposure to PM10, PM2.5–10, PM2.5, and incident infertility (primary and secondary)	Incident infertility	Living closer to major roadways and higher cumulative exposure to PM10 and PM2.5–10 were associated with increased risk of infertility, particularly secondary infertility.
Mendola et al. [17]	2017	Prospective cohort	393 couples attempting pregnancy from the LIFE study	Proximity to major roadways, time-to-pregnancy, and infertility (12 months without conception)	Fecundability and infertility	Couples living closer to major roadways (<1000 m) showed reduced fecundability and increased risk of infertility, with a 3% increase in pregnancy likelihood for every 200 meters further from a major roadway. The association, however, was not statistically significant.
Mohallem et al. [18]	2005	Experimental study	104 female Balb/c mice (divided into four groups)	Exposure to polluted air vs. filtered air, reproductive parameters (number of live-born pups, implantation failures, reabsorptions, and fetal deaths)	Reproductive outcomes in exposed vs. control mice	Mice exposed to air pollution from an early age had fewer live-born pups and a higher incidence of implantation failures compared to those in a clean environment. No significant differences were observed in other reproductive parameters.
Januário et al. [19]	2010	Experimental study	Swiss albino mice embryos	DEP concentrations, in vitro embryo development, blastocyst formation, apoptosis rates, Oct-4, and Cdx-2 expression	Embryo development, blastocyst quality, apoptosis, and gene expression	DEP exposure impaired early embryo development and blastocyst quality in a dose-dependent manner. Even low concentrations of DEP disrupted cell lineage specification and increased apoptosis.
Nieuwenhuijsen et al. [20]	2014	Cross-sectional study	Census tracts in Barcelona, Spain (2011-2012)	Fertility rates (general fertility rate, crude birth rate) and air pollution (PM10, PM2.5, PM2.5 absorbance, NO_2_, NOx, PMcoarse)	General fertility rate and crude birth rate	Higher levels of PMcoarse were significantly associated with reduced fertility rates in Barcelona, particularly in areas with high-traffic-related pollution. The association was most significant for PMcoarse, with an inverse relationship indicating that fertility rates decreased as PMcoarse levels increased.
Ogliari et al. [21]	2013	Experimental study	Swiss mice (exposed vs. control groups)	Exposure to diesel exhaust (intrauterine and postnatal), ovarian and uterine morphometry	Ovarian follicle counts and uterine morphology	Intrauterine exposure to diesel exhaust significantly reduced the proportion of primordial and primary follicles in adult mice, suggesting a diminished ovarian reserve. Postnatal exposure also reduced primary follicle proportion. No significant changes were detected in uterine morphology.
Perin et al. [22]	2010	Retrospective cohort study	400 first IVF/ET cycles	Ambient PM10 exposure during follicular phase, clinical and laboratory outcomes, and pregnancy outcomes	Pregnancy rates and miscarriage	No effect on clinical, laboratory, or treatment outcomes was observed, but exposure to high levels of ambient PM10 during the follicular phase increased the risk of miscarriage.
Quraishi et al. [23]	2019	Retrospective cohort	7,463 women undergoing IVF in the United States	Exposure to PM2.5, PM10, NO_2_, proximity to roadways, and IVF outcomes (pregnancy, live birth)	Pregnancy and live birth rates	A near-roadway residence was associated with decreased live birth rates. High exposure to PM2.5, PM10, and NO_2_ had a nonsignificant association with decreased live birth and increased pregnancy loss, particularly among women with diminished ovarian reserve or male infertility.
Slama et al. [24]	2013	Prospective cohort	1,916 couples from Teplice, Czech Republic	Exposure to PM2.5, NO_2_, SO_2_, carcinogenic PAHs, and ozone before pregnancy	Probability of pregnancy during the first month of unprotected intercourse	A 10 µg/m³ increase in PM2.5 was associated with a 22% decrease in fecundability. NO_2_ levels were also linked to decreased fecundability. No significant effects were observed for other pollutants.
Veras et al. [25]	2008	Experimental study	Inbred Balb/C mice (second-generation)	Exposure to filtered vs. nonfiltered urban air, placental volumes, surface areas, vessel diameters, and fetal weights	Placental morphology and fetal weight	Exposure to urban air pollution reduced fetal weight and altered placental morphology, particularly maternal blood space volumes and diameters, indicating compromised placental function and impaired fetal growth.
Wesselink et al. [26]	2019	Prospective cohort	10,183 women trying to conceive	Exposure to PM2.5, PM10, NOx, NO_2_, CO, O_3_, SO_2_; time-to-pregnancy, and fecundability ratio	Fecundability and time-to-pregnancy	Higher residential concentrations of PM2.5 and PM10 were associated with slightly reduced fecundability. The associations were stronger for short-term exposures during the menstrual cycle compared to long-term exposures.
Yang et al. [27]	2020	Experimental study	Male C57Bl/6J mice	Chronic exposure to diesel exhaust PM2.5, sperm count, motility, meiotic progression, testicular histology, and gene expression	Sperm count, motility, and meiotic progression	Chronic exposure to diesel exhaust PM2.5 significantly reduced sperm count and motility, impaired meiotic progression, and altered testicular gene expression, particularly affecting the repair of meiotic DSBs.
Zhu et al. [28]	2018	Retrospective cohort	53,094 singleton live births in Georgia	Exposure to PM2.5 (air quality index, AQI), maternal demographics, and preterm birth rates	Preterm birth rates	Mothers exposed to PM2.5 with AQI > 50 during pregnancy had a 15% higher risk of preterm birth compared to those exposed to lower AQI levels. The association was consistent after adjusting for potential confounders.
Padula et al. [29]	2012	Retrospective cohort	237,031 full-term singleton births in San Joaquin Valley, California	Traffic density near maternal residence, birth weight, maternal demographics	Term LBW	Higher traffic density during pregnancy was associated with an increased probability of term LBW. Specifically, living near high-volume freeways was associated with a 2.27% probability of term LBW compared to 2.02% for those living near smaller roads.
Padula et al. [30]	2014	Retrospective cohort	42,904 singleton births in Fresno, California	Exposure to airborne PAHs during pregnancy (measured via spatiotemporal model), preterm birth, and gestational age	Preterm birth and gestational age	Exposure to high levels of airborne PAHs during the last six weeks of pregnancy was associated with an increased risk of early preterm birth (20-27 weeks). The risk increased with higher PAH exposure levels. There were inverse associations with PAH exposure during the entire pregnancy and first trimester.
Díaz et al. [31]	2016	Time-series analysis	298,705 births in Madrid (2001–2009)	PM2.5, NO_2_, O_3_ concentrations, noise levels (Leqd, Leqn), and temperature	LBW in non-premature infants	PM2.5 exposure in the third month of pregnancy was associated with an increased risk of LBW. Noise levels, particularly diurnal noise (Leqd), were also associated with increased LBW risk during the week of birth. Temperature extremes (heat and cold) did not show a significant effect on LBW in non-premature births.
Cheng et al. [32]	2023	Observational study	70,854 singletons with gestational age <20 weeks in Wuhan, China	Exposure to PM10, PM2.5, NO_2_, SO_2_ during the first trimester; incidence of birth defects	Total birth defects, CHDs, limb defects, and orofacial clefts	Exposure to high concentrations of PM10, PM2.5, NO_2_, and SO_2_ during the first trimester significantly increased the odds of birth defects. The effects were stronger during the cold season and for male fetuses, particularly for CHDs associated with PM2.
Chen et al. [33]	2020	Observational study	282 infertile men in Taiwan	Exposure to SO_2_, NOx, PM2.5, O_3_; testicular volume, sperm quality, and sex hormone levels	Testicular volume, sperm concentration, and motility	Higher ambient SO_2_ exposure was associated with a significant decrease in testicular volume. NOx exposure was negatively associated with sperm concentration and motility.

Impact of wildfires

Impact of Wildfire Smoke on Preterm Birth and Birth Weight

The study by Abdo et al. [34] in Colorado linked exposure to wildfire smoke PM2.5 during the second trimester with an increased risk of preterm birth. Exposure during the first trimester was associated with decreased birth weight. Additionally, exposure to wildfire smoke increased the risks of gestational diabetes and hypertension. Holstius et al. [35] found that exposure to wildfire smoke during pregnancy in Southern California led to modest reductions in birth weight, especially during the second and third trimesters, with reductions of 9.7 and 7.0 grams, respectively. Heft-Neal et al. [36] showed that each additional day of wildfire smoke exposure during pregnancy increased the risk of preterm birth by 0.49%, with the second trimester being particularly vulnerable. Approximately 3.7% of preterm births during the study period were attributed to wildfire smoke exposure. Padula et al. [37] indicated that exposure to wildfire smoke, especially during the second and third trimesters, significantly increased the risks of preterm birth and LBW in California.

Epigenetic Effects and Sperm DNA Methylation

Schuller et al. [38] explored the impact of chronic exposure to simulated wildfire smoke on sperm DNA methylation in male mice. The findings revealed significant changes in DNA methylation, which were broadly distributed across the genome and associated with genes involved in developmental processes. This suggests that prolonged wildfire smoke exposure could have epigenetic effects on sperm, potentially leading to transgenerational reproductive health issues.

Wildfire Smoke and Pregnancy Outcomes in Animal Models

The study by Willson et al. [39] on non-human primates during the 2018 Camp Fire in Northern California found a significant increase in pregnancy loss among female rhesus macaques exposed to high levels of PM2.5 from wildfire smoke. The pregnancy loss rate in the exposed group was nearly double that of the control cohort.

Impact of heat

Impact of High Temperatures on Birth Rates

The study by Barreca et al. [41] analyzed U.S. birth rates from 1931 to 2010, and found that days with mean temperatures above 80°F led to a decrease in birth rates eight to 10 months later. The study suggests that high temperatures disrupt reproductive health, and the widespread adoption of air conditioning helped mitigate these negative effects.

Heat Stress and Stillbirths

The study by Rammah et al. [46] examined the relationship between high ambient temperatures and stillbirths in Texas. It found that a 10°F increase in temperature during the week before delivery significantly increased the risk of stillbirth, particularly among Hispanic and non-Hispanic Black women. Placental abruption was identified as a major factor in this increased risk.

Ambient Temperature and Ovarian Reserve

Gaskins et al. [43] explored the relationship between ambient temperature and ovarian reserve in women, finding that higher temperatures in the three months before testing were associated with lower AFC, indicating that increasing temperatures could accelerate reproductive aging in women.

Heat and Placental Abruption

The study by He et al. [44] found that elevated ambient temperatures (30°C or higher) during the warm season in Quebec increased the risk of placental abruption at term, especially among younger and socioeconomically disadvantaged women. The study emphasizes the vulnerability of pregnant women to heat stress as they approach term.

Maternal Hyperthermia and Neural Tube Defects (NTDs)

The study by Suarez et al. [47] found that maternal hyperthermia due to fever or external heat exposures during the first trimester significantly increased the risk of NTDs in a Mexican-American population. The study suggests the importance of managing febrile illnesses and avoiding heat exposures during early pregnancy to reduce the risk of congenital defects.

Impact of Heat Stress on Reproductive Health in Animal Models

The study by Pérez-Crespo et al. [42] in a mouse model found that scrotal heat stress significantly reduced sperm viability, motility, and DNA integrity, with lasting effects on male fertility. The study also noted a shift in the sex ratio of offspring, with fewer males being born when heat-stressed sperm were used, suggesting that heat stress may differentially affect X- and Y-chromosome-bearing sperm.

Heat Stress and Dairy Cow Fertility

The study by Pavani et al. [45] on Holstein dairy cows showed that high temperatures, as indicated by the temperature humidity index (THI), were negatively correlated with conception rates, particularly during the summer. Heat stress also reduced the meiotic competence of oocytes, leading to lower success rates in IVF outcomes.

Impact of floods

The studies by Simcock et al. [48] and Ping et al. [49] both explored the effects of prenatal maternal stress (PNMS) due to natural disasters on early child development, emphasizing the significant impact of maternal psychological responses on children's outcomes. Simcock et al. [48] examined the 2011 Queensland floods' impact on children's theory of mind (ToM) at 30 months, finding that higher maternal subjective stress during the flood was linked to poorer ToM performance, especially in girls, where maternal cognitive appraisal played a moderating role. In contrast, Ping et al. [49] studied the 2008 Iowa floods, focusing on cortisol reactivity in toddlers. They found that higher subjective PNMS was associated with increased cortisol reactivity in female toddlers, particularly when stress occurred later in pregnancy, while male toddlers showed less cortisol reactivity. Both studies underscore the profound influence of maternal stress during pregnancy on children's social cognition and stress-response systems, highlighting the need for supportive interventions for pregnant women in disaster-affected regions to mitigate potential long-term developmental impacts on their children.

Impact of toxic chemicals and heavy metals

The study by Nishijo et al. [50] investigated the impact of maternal cadmium exposure on pregnancy outcomes and cadmium transfer to infants through breast milk. The study was conducted in Toyama, Japan, an area historically affected by cadmium pollution. Fifty-seven pregnant women were included in the study, and their urinary cadmium levels were measured as an indicator of body burden. The findings revealed that higher urinary cadmium levels in pregnant women were significantly associated with increased rates of preterm birth and LBW. Additionally, infants born to mothers with higher urinary cadmium levels also had higher cadmium concentrations in their breast milk. This study suggests that cadmium exposure during pregnancy can adversely affect birth outcomes and cadmium can be transferred to infants through breast milk, potentially posing additional health risks to newborns. Table 2 summarizes the studies regarding the impact of wildfires, heat, floods, toxic chemicals, and heavy metals.

**Table 2:** Summary of studies included in the systematic review regarding the impact of wildfires, heat, floods, toxic chemicals, and heavy metals AFC: Antral follicle count; BMI: Body mass index; CO: Carbon monoxide; DNA: Deoxyribonucleic acid; HS: Heat shock; MAFT: Missed abortion in the first trimester; NO_2_: Nitrogen dioxide; NTD: Neural tube defects; O_3_: Ozone; PM2.5: Particulate matter less than 2.5 micrometers; PNMS: Prenatal maternal stress; RRBS: Reduced representation bisulfite sequencing; SO_2_: Sulfur dioxide; THI: Temperature humidity index; ToM: Theory of mind.

Authors	Year	Type of study	Sample	Measurements	Outcomes	Key findings
Abdo et al. [34]	2019	Retrospective cohort	535,895 singleton births in Colorado	Exposure to wildfire smoke PM2.5 (estimated using remote sensing and ground-based monitors), birth outcomes (preterm birth and birth weight), gestational age, and maternal demographics	Preterm birth, low birth weight, gestational diabetes, and gestational hypertension	Wildfire smoke PM2.5 exposure during the second trimester was significantly associated with an increased risk of preterm birth, and first-trimester exposure was linked to decreased birth weight. There were also significant associations with gestational diabetes and hypertension.
Holstius et al. [35]	2012	Retrospective cohort	886,034 term births in Southern California	Exposure to wildfires during pregnancy (by trimester), birth weight, and maternal demographics	Birth weight	Exposure to wildfire smoke during pregnancy was associated with a slight reduction in birth weight, particularly when exposure occurred during the second trimester (9.7 g lower on average) and third trimester (7.0 g lower on average).
Heft-Neal et al. [36]	2021	Retrospective cohort	3,002,014 singleton births in California	Exposure to wildfire smoke (smoke days, PM2.5 intensity), maternal demographics, and preterm birth	Preterm birth (<37 weeks)	Each additional day of wildfire smoke exposure was associated with a 0.49% increase in preterm birth risk. The effect was strongest during the second trimester and high-intensity smoke days. Approximately 3.7% of preterm births during the study period were attributable to wildfire smoke exposure.
Padula et al. [37]	2014	Retrospective cohort	325,717 live births in California	Exposure to wildfire smoke PM2.5 (measured by satellite and ground-based monitors), maternal demographics, and pregnancy outcomes	Preterm birth, low birth weight, and stillbirth	Exposure to wildfire smoke during pregnancy, particularly in the second and third trimesters, was associated with increased risks of preterm birth and low birth weight. The association was strongest for mothers exposed to high levels of PM2.5 during late pregnancy.
Schuller et al. [38]	2021	Experimental study	Male Apoe−/− mice	Chronic exposure to simulated wildfire smoke (Douglas fir needles) and sperm DNA methylation (via RRBS)	DNA methylation patterns and gene ontology	Wildfire smoke exposure significantly altered sperm DNA methylation, with 79% of differentially methylated regions showing hypermethylation. Genes related to developmental processes were particularly affected, indicating potential reproductive risks associated with prolonged smoke exposure.
Wilson et al. [39]	2021	Case-control study	66 reproductive-age female rhesus macaques	Exposure to PM2.5 from the Camp Fire, conception rates, live birth rates, and pregnancy loss	Pregnancy loss and live birth rates	Exposure to elevated PM2.5 levels during the 2018 Camp Fire was associated with a significant increase in pregnancy loss in rhesus macaques. The study found that the pregnancy loss rate in the exposed cohort was nearly double that of the control cohort from previous years.
Zhang et al. [40]	2019	Retrospective cohort	255,668 pregnant women in Beijing	Exposure to PM2.5, SO_2_, O_3_, CO; missed abortion (MAFT) risk	Missed abortion in the first trimester (MAFT)	Increased risk of MAFT was associated with higher concentrations of air pollutants. For example, a 10 µg/m³ increase in PM2.5 was linked to a 51% higher risk of MAFT at concentrations >130.2 µg/m³. Similar risk increases were observed for SO_2_, O_3_, and CO, with risk growing more severe at higher pollutant concentrations.
Barreca et al. [41]	2018	Retrospective cohort study	Birth records from the United States from 1931 to 2010	Daily mean temperature data, birth rates by month, state-level temperature, and fertility analysis	Birth rate changes, conception timing	Exposure to days with temperatures above 80°F leads to a significant decline in birth rates 8-10 months later, with a partial rebound in the following months. The study suggests that high temperatures affect reproductive health rather than sexual activity. Access to air conditioning mitigates these effects.
Pérez-Crespo et al. [42]	2008	Experimental study	Male CD1 mice	Exposure to scrotal heat (42°C for 30 minutes), sperm viability, DNA integrity, and offspring sex ratio	Sperm viability, motility, DNA integrity, and offspring sex ratio	Scrotal heat stress significantly reduced sperm viability, motility, and DNA integrity, particularly 14 days post-exposure. The sex ratio of offspring was also distorted, with a lower proportion of males when heat-exposed spermatozoa were used for fertilization immediately after stress.
Gaskins et al. [43]	2021	Prospective cohort	631 women attending a fertility clinic	The ambient temperature at a residential address, antral follicle count (AFC)	Ovarian reserve (measured as AFC)	A 1°C increase in average maximum temperature during the 90 days prior to ovarian reserve testing was associated with a 1.6% lower AFC. This suggests that exposure to higher temperatures may accelerate reproductive aging in women. The effect was stronger outside the summer months, indicating possible acclimatization during summer.
He et al. [44]	2017	Case-crossover study	17,172 pregnancies in Quebec, Canada	Maximum weekly temperature, relative humidity, public holidays, gestational age, and maternal demographics	Placental abruption at term vs. preterm	Elevated temperatures (≥30°C) were associated with a 12% higher risk of placental abruption at term. The risk was stronger in younger women, nulliparous women, and those socioeconomically disadvantaged. No significant association was found for preterm abruption.
Pavani et al. [45]	2015	Experimental study	6,300 Holstein cows and 1,597 oocytes	Temperature humidity index (THI), conception rates, oocyte meiotic maturation, and embryo development	Conception rates, oocyte maturation, and embryo development	THI was negatively correlated with conception rates, with lower rates during warmer months. Heat shock reduced meiotic maturation rates and embryo development, with a clear decline observed at higher temperatures (HS1: 39.5°C, HS2: 40.5°C).
Rammah et al. [46]	2019	Case-crossover study	1,599 stillbirths in Harris County, Texas	Apparent temperature, air pollutant levels (PM2.5, NO_2_, O_3_), and maternal demographics	Stillbirth and placental abruption	A 10°F increase in apparent temperature during the week preceding delivery was associated with a 45% increased risk of stillbirth, particularly during June to August. The risk was highest for stillbirths caused by placental abruption, with a nearly doubled risk on the day before delivery.
Suarez et al. [47]	2004	Case-control study	184 Mexican-American women with NTD-affected pregnancies, 221 controls	Maternal fever, febrile illnesses, heat exposures, folate intake, and BMI	Neural tube defects (NTDs)	Maternal fever during the first trimester increased the risk of NTDs (OR 2.9). Women who took fever-reducing medications had a lower risk compared to those who did not. Exposures to external heat sources (e.g., hot tubs, electric blankets) also significantly increased the risk of NTDs (OR 3.6).
Simcock et al. [48]	2017	Longitudinal study	130 mother-child pairs exposed to the 2011 Queensland floods during pregnancy	Maternal objective hardship, subjective stress, cognitive appraisal, and child theory of mind (ToM) at 30 months	Theory of Mind (ToM) performance in children	Higher maternal subjective stress during the flood was associated with poorer ToM performance in children at 30 months. Cognitive appraisal moderated these effects, with girls showing greater sensitivity to maternal cognitive appraisal than boys. Objective hardship was not a significant predictor.
Ping et al. [49]	2015	Longitudinal study	94 mother-toddler dyads affected by the 2008 Iowa floods	Objective and subjective prenatal stress measures and cortisol levels in toddlers	Cortisol reactivity in toddlers	Higher levels of prenatal maternal stress (PNMS), particularly subjective stress, were associated with increased cortisol reactivity in female toddlers at 2½ years of age. The effect was more pronounced with later exposure in pregnancy. Males showed less cortisol reactivity to maternal stress.
Nishijo et al. [50]	2002	Observational study	57 pregnant women in Toyama, Japan	Urinary cadmium concentration, breast milk cadmium concentration, birth weight, and gestational age	Preterm birth, birth weight, and cadmium levels in breast milk	Higher maternal urinary cadmium levels were associated with increased rates of preterm birth, lower birth weight, and higher cadmium concentrations in breast milk. There was a significant correlation between urinary cadmium levels and cadmium levels in breast milk.
Kwag et al. [51]	2021	Retrospective cohort study	1.33 million births in Korea (2010-2016)	PM2.5 concentration, heat wave exposure, and gestational age at birth	Preterm birth (<37 weeks)	Heatwave exposure during the second trimester significantly increased the risk of preterm birth, with a combined effect observed when PM2.5 levels were also high. The odds of preterm birth were highest for those exposed to >315 hours of heatwave and higher quartiles of PM2.5 during the second trimester

Vector-borne infectious diseases

The studies on dengue fever during pregnancy underscore the severe risks and adverse outcomes associated with the infection. Brar et al. [52] conducted a study in India that revealed a high maternal mortality rate (15.9%) among pregnant women with dengue, along with significant complications such as preterm birth (34.1%), LBW (29.5%), postpartum hemorrhage (25%), and maternal conditions like acute kidney injury and acute respiratory distress syndrome. Similarly, Paixão et al. [53] in Brazil found that dengue hemorrhagic fever during pregnancy doubled the risk of preterm birth and LBW, with even mild dengue increasing these risks by 10%-20%. Martin et al. [54] further emphasized the heightened vulnerability of pregnant women, demonstrating that they had a nearly threefold higher risk of hospitalization and a more than fivefold increased risk of developing severe dengue compared to non-pregnant women.

The study by Hoen et al. [56] conducted in French territories in the Americas found that 7% of fetuses and infants exposed to ZIKV during pregnancy developed neurologic and ocular defects, with a higher risk when the infection occurred in the first trimester, including a 5.8% incidence of microcephaly. Similarly, the research by Rosado et al. [55] in Central-West Brazil during a post-epidemic period reported that 7% of ZIKV-exposed children had microcephaly, and 11.4% developed congenital Zika syndrome (CZS), with 54.5% showing ophthalmological abnormalities. Both studies underscore the significant risks of congenital abnormalities and adverse pregnancy outcomes associated with ZIKV infection, particularly when contracted early in pregnancy, and highlight the importance of long-term monitoring of affected infants due to potential delayed-onset complications.

The studies on malaria during pregnancy consistently highlight the significant risks associated with the infection, particularly in terms of adverse birth outcomes and infant health. Bardají et al. [58] found that malaria during late pregnancy in southern Mozambique significantly increased the risk of infant mortality and malaria morbidity in the first year of life. Similarly, Hussein et al. [59] demonstrated that prenatal malaria exposure in the third trimester in Ghana was associated with higher odds of preterm birth and LBW, but not with increased perinatal mortality. In Mali, Gaoussou et al. [60] reported that malaria heightened the risk of preterm delivery, especially in first-time mothers and intermittent preventive treatment during pregnancy with sulfadoxine-pyrimethamine (IPTp-SP) reduced adverse outcomes like stillbirth and neonatal death. Lastly, Lufele et al. [57] in Papua New Guinea identified placental malaria in 18.5% of pregnant women, linking acute infections to LBW and chronic infections to preterm delivery and maternal anemia. Collectively, these studies emphasize the urgent need for effective malaria prevention and treatment strategies during pregnancy to mitigate these risks, particularly in malaria-endemic regions. Table 3 summarizes the studies regarding the impact of vector-borne infectious diseases.

**Table 3:** Summary of studies included in the systematic review regarding the impact of vector-borne infectious diseases CZS: Congenital Zika syndrome; DENV: Dengue virus; IgM: Immunoglobulin M; IPTp-SP: Intermittent preventive treatment in pregnancy with sulfadoxine-pyrimethamine; PRNT90: Plaque reduction neutralization test 90; PTD: Preterm delivery; RT-PCR: Reverse transcription polymerase chain reaction; SGA: Small for gestational age; ZIKV: Zika virus.

Authors	Year	Type of study	Sample	Measurements	Outcomes	Key findings
Brar et al. [52]	2021	Prospective observational study	44 pregnant women with dengue fever	Clinical symptoms, platelet counts, liver function tests, renal function tests, and coagulation profiles	Maternal mortality, preterm birth, low birth weight, and postpartum hemorrhage	Dengue fever during pregnancy was associated with high maternal mortality (15.9%), high rates of postpartum hemorrhage (25%), preterm births (34.1%), and low birth weight (29.5%). The study also reported a significant incidence of maternal complications, including acute kidney injury and acute respiratory distress syndrome.
Paixão et al. [53]	2019	Population-based cohort study	16,738,000 live births in Brazil (2006–2012)	Exposure to dengue (mild, complicated, hemorrhagic), gestational age, birth weight, and SGA	Preterm birth, low birth weight, and SGA	Dengue hemorrhagic fever in pregnancy was associated with a doubled risk of preterm birth and low birth weight. Mild dengue increased the risk of preterm birth and low birth weight by 10%-20%. There was no significant association with SGA. The effects were strongest during the acute phase of the disease.
Martin et al. [54]	2022	Retrospective cohort study	27,605 women of reproductive age (949 pregnant and 26,656 non-pregnant)	Clinical data, hospitalization records, and severity classification (dengue, dengue with warning signs, severe dengue)	Severity of dengue infection, hospitalization, laboratory testing, and identification of DENV serotypes	Pregnancy was associated with a higher risk of hospitalization and severe dengue; increased monitoring and treatment are necessary for pregnant women.
Rosado et al. [55]	2023	Prospective cohort study	81 pregnant women in Central-West Brazil	Zika virus infection confirmed by RT-PCR, IgM, PRNT90, birth outcomes, ophthalmological, and neurological assessments	Microcephaly, congenital Zika syndrome, ophthalmological, and neurological abnormalities	7% of ZIKV-exposed children had microcephaly; 11.4% had CZS; 54.5% of ZIKV-exposed children had ophthalmological abnormalities, including chorioretinal atrophy and optic nerve hypoplasia.
Hoen et al. [56]	2018	Prospective cohort study	546 pregnant women in French territories (Guadeloupe, Martinique, and French Guiana)	ZIKV infection confirmed by RT-PCR, birth outcomes, and neurologic and ocular defects	Birth defects, miscarriage, and stillbirth	7% of fetuses and infants had neurologic and ocular defects possibly associated with ZIKV infection. The risk of birth defects was higher when infection occurred during the first trimester. Microcephaly was detected in 5.8% of fetuses and infants.
Lufele et al. [57]	2017	Prospective cohort study	1,451 pregnant women in Papua New Guinea	Placental malaria infection status, birth weight, maternal hemoglobin, and preterm delivery	Low birth weight, preterm delivery, and maternal anemia	Placental malaria was detected in 18.5% of women. Acute infections were associated with low birth weight, while chronic infections were linked to preterm delivery and maternal anemia. Risk factors for placental malaria included rural residence, primigravidity, and symptomatic malaria during pregnancy.
Bardají et al. [58]	2011	Randomized, placebo-controlled trial	1,030 pregnant women in Mozambique	Malaria infection status, birth outcomes, infant mortality, and clinical malaria episodes in infants	Infant mortality, malaria, and morbidity in infants	Maternal malaria infection at the end of pregnancy was associated with increased infant mortality and higher rates of malaria morbidity during infancy. Acute placental infection and cord blood parasitemia were strongly associated with these adverse outcomes.
Hussein et al. [59]	2022	Prospective cohort study	1,323 pregnant women in the northern region of Ghana	Malaria exposure during the third trimester (diagnosed via a rapid diagnostic test) and birth outcomes (low birth weight, preterm birth, perinatal death)	Low birth weight, preterm birth, and perinatal death	Women exposed to malaria during the third trimester had 2.02 times higher odds of preterm delivery and 2.06 times higher odds of low birth weight. No significant difference in perinatal mortality was observed after adjusting for confounders.
Gaoussou et al. [60]	2022	Observational study	505 women of child-bearing age in Mali	Monthly pregnancy tests, malaria infection status, and antenatal care visits	Miscarriage, stillbirth, preterm delivery, neonatal death, and SGA	The study identified a high rate of miscarriage (12%) in early pregnancy, particularly in women over 35 and those with a previous miscarriage. Malaria infection increased the risk of PTD, especially in primigravidae. IPTp-SP reduced the risk of adverse outcomes.

Discussion

The review consistently underscores the significant impact of environmental factors on reproductive health, particularly with proximity to major roadways, exposure to air pollutants, and extreme temperatures. Women living near high-traffic areas demonstrated lower success rates in IVF, reduced implantation and live birth rates, and a higher risk of infertility, likely due to chronic exposure to traffic-related pollutants and noise. Furthermore, exposure to particulate matter, especially PM2.5, has been linked to decreased fecundability, lower ovarian reserve, and an increased risk of miscarriage. The detrimental effects extend to male fertility as well, with studies indicating that air pollution adversely affects sperm quality, leading to reduced motility and sperm count. These findings highlight the critical need for addressing environmental pollution to protect reproductive health.

Exposure to wildfire smoke during pregnancy is associated with increased risks of preterm birth and LBW, while the expansion of disease vectors due to climate change poses a growing threat of infections like ZIKV, which can lead to miscarriage and congenital anomalies. Moreover, there are potential transgenerational effects on reproductive health. The compounded risks posed by psychosocial stress are also emphasized, particularly in the wake of natural disasters, which can exacerbate adverse pregnancy outcomes and affect the long-term developmental health of offspring.

The impacts of climate change and its cascading effects disproportionately affect women, particularly those of lower socioeconomic status, pregnant women and their developing fetuses, the elderly, the disabled, and young children [61]. Women are often the primary caregivers in households, responsible for childcare, elder care, and other family needs. They are also tasked with managing essential household resources like water, energy for cooking, heating, and food preparation. Historical gender inequalities in reproductive rights, political and economic status, education, and legal protection are exacerbated during environmental crises [61]. Sociocultural norms and family obligations may prevent women from migrating out of high-risk areas, making them particularly vulnerable during times of societal breakdown or family structure deterioration. Women are at an increased risk of gender-based and domestic violence, sexual intimidation, human trafficking, and rape and are 14 times more likely than men to die in disasters [62]. Their limited socioeconomic power hinders disaster recovery, affecting infrastructure, job access, and suitable housing. In many societies, women have minimal political decision-making power and are rarely involved in environmental management. Recognizing women as a vulnerable population highlights their risks from climate change and its consequences and also emphasizes the importance of involving them in mitigation strategies due to their unique experiences [63].

The review consistently highlights the significant impact of environmental factors on reproductive health, particularly among women living near high-traffic areas and those exposed to air pollutants and extreme temperatures. However, to fully understand the disparities in reproductive health outcomes, it is essential to consider the intersection of environmental and sociocultural factors. Women, especially those from lower socioeconomic backgrounds, often bear a disproportionate burden of environmental risks due to pre-existing gender inequalities [64].

Sociocultural factors such as limited access to healthcare, lower economic status, and restrictive gender norms exacerbate women's vulnerability to environmental risks. For instance, women in disaster-prone areas may have limited mobility due to childcare responsibilities or cultural expectations, making it difficult for them to seek shelter or medical assistance during extreme weather events. This compounded exposure to both environmental and sociocultural risks significantly increases the likelihood of adverse reproductive health outcomes, such as miscarriage, preterm birth, and low birth weight [65].

Moreover, the stress induced by sociocultural pressures, such as economic instability and gender-based violence, can exacerbate the physiological impacts of environmental exposures. For example, pregnant women experiencing high levels of psychosocial stress may be more susceptible to the harmful effects of air pollution or heat waves, leading to worse pregnancy outcomes. The cumulative effect of these intersecting risks underscores the need for comprehensive public health strategies that address both environmental and sociocultural determinants of reproductive health. Addressing these intersections requires not only environmental interventions but also social policies that empower women, improve access to healthcare, and mitigate the sociocultural barriers that increase their vulnerability [66].

Cramped living spaces with inadequate ventilation expose them to higher levels of indoor pollutants, exacerbating respiratory issues and stress, which are particularly harmful during pregnancy. Limited access to quality healthcare, including essential prenatal care, hinders early intervention for complications, increasing the likelihood of adverse outcomes such as preterm birth and LBW. Additionally, women in lower-wage manual jobs are often exposed to harmful chemicals and pollutants, including endocrine-disrupting chemicals, which can interfere with hormone regulation during pregnancy. The psychosocial stress induced by economic instability further compounds these risks, making these women more vulnerable to issues like miscarriage and preterm labor [67].

Women living in disaster-prone areas face significant reproductive health risks due to increased exposure to environmental hazards such as floods, hurricanes, and wildfires, which can elevate the likelihood of preterm birth, LBW, and other complications. Disasters frequently lead to displacement, disrupting access to essential healthcare services, including prenatal care, and the associated stress and physical challenges further threaten pregnancy outcomes. Additionally, cultural and mobility restrictions in these regions may limit women’s ability to evacuate or seek timely medical assistance, exacerbating their vulnerability and increasing the risk of adverse reproductive health outcomes [68].

Various aspects of climate change, such as exposure to endocrine disruptors like PAHs, Cu, Ph, and Zn in particulate matter from diesel exhaust, have adverse effects on reproductive health. These disruptors can induce reactive oxygen species, leading to DNA damage, epigenetic changes, abnormal DNA repair, and disruptions in normal gene/protein expression and cellular and organ function. Air pollution, particularly PM2.5 and O_3_, along with heat exposure, is linked to preterm birth, LBW at term, and stillbirth [51,69]. The most at-risk groups include Black women, Hispanic women, and those with asthma. In Japan, temperature fluctuations due to climate change have been associated with an increase in male stillbirths, though the mechanisms are uncertain [23]. Certain components of air pollution may work synergistically to exacerbate health effects through oxidative stress, immune, and inflammatory pathways. For instance, higher temperatures and drought conditions increase the prevalence of wildfires. A study in Australia found that exposure to PM2.5 had an additive effect during heatwaves, increasing emergency room visits by 6.6% [70]. Additionally, air pollution exposure has been linked to higher mortality rates from COVID-19, contributing to approximately 15% of global deaths from the virus [71]. Some hypothesize that air pollution and COVID-19 may also synergistically reduce sperm counts through pro-inflammatory pathways involving the testicular angiotensin-converting enzyme 2 receptor [72].

The findings from this systematic review underscore the urgent need for public health interventions and policies aimed at mitigating environmental exposures that negatively impact reproductive health. Given the significant associations between air pollution, extreme temperatures, and adverse reproductive outcomes, there is a compelling case for stricter environmental regulations [73]. This includes enforcing air quality standards, especially in urban areas, and implementing policies to protect vulnerable populations, such as pregnant women, from the harmful effects of pollution and extreme weather events [74]. Additionally, public health campaigns should focus on raising awareness about the risks of environmental exposures and promoting behaviors that reduce these risks, such as avoiding high-pollution areas during pregnancy [75].

Strengths of the systematic review

This systematic review provides a comprehensive evaluation of the impact of environmental factors, particularly those related to climate change, on reproductive health and pregnancy outcomes. The review is robust in its methodology, employing a rigorous search strategy across multiple databases and adhering to the PRISMA guidelines to ensure a transparent and reproducible study selection process. The inclusion of a diverse range of study designs, including prospective cohort studies, RCTs, case-control studies, cross-sectional studies, and experimental animal studies, enhances the generalizability and robustness of the findings. Additionally, the use of standardized tools for quality assessment, such as the NOS and Cochrane Risk of Bias Tool, ensures that the included studies are critically appraised, thereby minimizing bias and increasing the reliability of the conclusions drawn. Another significant strength of this review is its global scope, which includes studies from various geographic regions, allowing for a broad understanding of how different environmental exposures might impact reproductive health across diverse populations. This global perspective is crucial in the context of climate change, where environmental impacts can vary widely by region. Moreover, the review addresses a wide range of environmental exposures, including air pollution, temperature extremes, and vector-borne diseases, providing a holistic view of how climate change-related factors can affect reproductive outcomes.

Limitations of the systematic review

Despite its strengths, this systematic review also has several limitations. One major limitation is the heterogeneity of the included studies in terms of their exposure assessment methods, outcome measures, and population characteristics. This variability can complicate the synthesis of findings and may limit the ability to draw firm conclusions about specific environmental exposures. Additionally, the reliance on observational studies, which are subject to confounding factors and cannot establish causality, limits the strength of the evidence. While experimental studies in animals provide valuable insights, their applicability to human populations may be limited due to species differences.

Another limitation is the potential for publication bias, as the review only included studies published in English and available as full-text articles. This exclusion criteria might have resulted in the omission of relevant studies published in other languages or as abstracts, potentially skewing the findings. Furthermore, the review's focus on mammalian species means that other relevant research on non-mammalian models was not considered, which could have provided additional insights into the mechanisms underlying the observed effects. Lastly, the review's reliance on studies up to a certain date may have missed more recent research developments, particularly in the rapidly evolving field of climate change and health. As the effects of climate change continue to intensify, ongoing research is likely to yield new findings that could further inform and refine the conclusions drawn in this review.

Suggestions for future research

Future research should aim to address the limitations identified in this review. This includes conducting more longitudinal and interventional studies to better establish causal relationships between environmental exposures and reproductive outcomes. There is also a need for more standardized and precise exposure assessment methods to reduce variability and improve the comparability of studies.

Additionally, future research should expand to include non-English studies and those from underrepresented regions, ensuring a more comprehensive understanding of the global impact of climate change on reproductive health. Studies exploring the mechanistic pathways by which environmental factors affect reproductive outcomes will also be crucial in developing targeted interventions.

Lastly, given the evolving nature of climate change and its impacts, researchers need to continue monitoring and updating the evidence base to inform effective public health responses and policy decisions.

## Conclusions

The findings of this systematic review reveal a clear and concerning link between climate change and adverse reproductive health outcomes. The evidence demonstrates that environmental factors associated with climate change, including air pollution and extreme temperatures, significantly impact fertility, pregnancy outcomes, and fetal development. Vulnerable populations, particularly women of lower socioeconomic status and those in disaster-prone areas, are disproportionately affected. These findings highlight the critical need for comprehensive public health strategies and policies aimed at reducing environmental exposures and supporting vulnerable groups. As climate change continues to intensify, ongoing research and proactive interventions will be essential to safeguarding reproductive health globally.
